# A Search for New Biological Pathways in Cerebral Autosomal Dominant Arteriopathy with Subcortical Infarcts and Leukoencephalopathy by Proteomic Research

**DOI:** 10.3390/jcm13113138

**Published:** 2024-05-27

**Authors:** Paloma Menéndez-Valladares, Rosa Acevedo Aguilera, David Núñez-Jurado, Cristina López Azcárate, Ana María Domínguez Mayoral, Alejandro Fernández-Vega, Soledad Pérez-Sánchez, Marcel Lamana Vallverdú, María Isabel García-Sánchez, María Morales Bravo, Teresa Busquier, Joan Montaner

**Affiliations:** 1 Department of Neurology, Virgen Macarena University Hospital, 41009 Seville, Spain; palomamenval@gmail.com (P.M.-V.); rosa.acevedo.aguilera@gmail.com (R.A.A.); dvdnj87@gmail.com (D.N.-J.); lopezcristinafisevi@gmail.com (C.L.A.); soledad.perez.sanchez@gmail.com (S.P.-S.); marcel.lamana@gmail.com (M.L.V.); mariamoralesbravo21@gmail.com (M.M.B.); jmontaner-ibis@us.es (J.M.); 2 Department of Neurology, Institute of Biomedicine of Seville (IBIS), 41013 Seville, Spain; 3 Department of Clinical Biochemistry, Virgen Macarena University Hospital, 41009 Seville, Spain; 4 Commission of Neurochemistry and Neurological Diseases, Spanish Society of Laboratory Medicine, 08025 Barcelona, Spain; 5 Virgen Macarena Hospital Biobank, Biobank of the Public Health System of Andalusia, 41009 Seville, Spain; mariaisabel.garcia.sanchez@juntadeandalucia.es; 6 Department of Radiology, Virgen Macarena University Hospital, 41009 Seville, Spain; tbusquier@gmail.com

**Keywords:** CADASIL, proteomics, small vessel disease, biomarkers, stroke

## Abstract

**Background/Objectives**: Cerebral Autosomal Dominant Arteriopathy with Subcortical Infarcts and Leukoencephalopathy (CADASIL) is a hereditary small vessel disease leading to significant morbidity and mortality. Despite advances in genetic diagnosis, the underlying pathophysiology remains incompletely understood. Proteomic studies offer insights into disease mechanisms by identifying altered protein expression patterns. Here, we conducted a proteomic analysis to elucidate molecular pathways associated with CADASIL. **Methods**: We enrolled genetically diagnosed CADASIL patients and healthy, genetically related controls. Plasma samples were subjected to proteomic analysis using the Olink platform, measuring 552 proteins across six panels. The data were analyzed from several approaches by using three different statistical methods: Exploratory Principal Component Analysis (PCA) and Partial Least Squares–Discriminant Analysis (PLS-DA), differential expression with moderated *t*-test, and gene set enrichment analysis (GSEA). In addition, bioinformatics analysis, including volcano plot, heatmap, and Variable Importance on Projection (VIP) scores from the PLS-DA model were drawn. **Results**: Significant differences in protein expression were observed between CADASIL patients and controls. RSPO1 and FGF-19 exhibited elevated levels (*p* < 0.05), while PPY showed downregulation (*p* < 0.05) in CADASIL patients, suggesting their involvement in disease pathogenesis. Furthermore, MIC-A/B expression varied significantly between patients with mutations in exon 4 versus exon 11 of the *NOTCH3* gene (*p* < 0.05), highlighting potential immunological mechanisms underlying CADASIL. We identified altered pathways using GSEA, applied after ranking the study data. **Conclusions**: Our study provides novel insights into the proteomic profile of CADASIL, identifying dysregulated proteins associated with vascular pathology, metabolic dysregulation, and immune activation. These findings contribute to a deeper understanding of CADASIL pathophysiology and may inform the development of targeted therapeutic strategies. Further research is warranted to validate these biomarkers and elucidate their functional roles in disease progression.

## 1. Introduction

Cerebral small vessel disease (CSVD) encompasses various neurological disorders affecting small arteries, capillaries, and veins, contributing to 20–30% of ischemic strokes and cerebral hemorrhages. It is associated with cognitive impairment and age-related disability [1]. The primary cause of CSVD is typically sporadic, often linked to hypertension, while less common cases involve genetic factors like CADASIL (Cerebral Autosomal Dominant Arteriopathy with Subcortical Infarcts and Leukoencephalopathy), the most prevalent hereditary form of the disease. Recent research suggests that disruptions in the cerebrovascular matrisome, which encompasses proteins forming the extracellular matrix and associated proteins, represent a common pathological pathway in both monogenic and sporadic forms of CSVD [2]. The emerging concept of the neurovascular unit highlights the close relationship between pathological processes in the brain’s microcirculation and parenchymal pathology [3].

CADASIL is an autosomal dominant cerebrovascular disease characterized by non-atherosclerotic and non-amyloid diffuse angiopathy, primarily affecting small to medium-sized penetrating and leptomeningeal arteries. It results from mutations in the NOTCH3 gene located on chromosome 19p13.2–p13.1 [4,5]. It is estimated to have an early prevalence of 2 to 5 cases per 100,000 worldwide. It is classified as a rare disease according to the European definition, affecting less than 1 person per 2000 [6]. However, this prevalence may be significantly underestimated, with a predicted mutation frequency of 4.1 to 10.7 carriers per 100,000 [7]. Predominant clinical features include migraines with aura (20–40%), recurrent strokes of ischemic nature (60–85%), psychiatric disturbances (25–30%), and cognitive impairment (60%), contributing to disability and life-threatening conditions [8]. While diagnosis primarily relies on clinical presentation, autosomal dominant inheritance, and distinctive brain magnetic resonance imaging (MRI) abnormalities, genetic testing confirming a pathogenic NOTCH3 mutation remains pivotal [5].

Furthermore, the deposition of granular osmiophilic material (GOM) in vascular smooth muscle cells (VSMCs) is a key pathological feature of arteriopathy in CADASIL [9]. GOM deposits contain various proteins, including NOTCH3 ectodomain and N-terminal fragment (NTF), inhibitor metalloproteinase 3 (TIMP3), and vitronectin (VTN) [10,11]. While the exact cause of CADASIL remains elusive, studies suggest aberrant NOTCH3 signaling, toxic aggregation of NOTCH3 extracellular domain (NECD), and matrisome alterations influenced by factors like hypertension and smoking [12]. NOTCH3 mutations likely lead to a gain-of-function effect, as evidenced by the upregulation of TIMP3 and VTN at sites of NOTCH3 aggregation [7]. Inflammatory and autoimmune mechanisms may contribute, with cytokines like ICAM-1 and IL-6 upregulated in VSMCs [13]. Several potential diagnostic and prognostic biomarkers, including TIMP3, VTN, NOTCH3, MIC-A/B, ICAM1, ANXA1, CXCL10, COL18A1, VEGFA, PTPRS, AREG, and SOD1, have been identified [14,15,16]. Despite these advancements, further research, particularly on blood samples, is needed to fully explore the diagnostic and prognostic potential of these proteins in CADASIL.

The rapid development of high-throughput omics technologies provides new avenues for dissecting pathophysiological mechanisms and discovering biomarkers in complex diseases. Proteomics, a widely used methodology for studying proteins produced in cells, tissues, and body fluids, offers significant promise for biomarker screening in human diseases [17]. Olink technology, known for its excellent reproducibility and stability, has been increasingly utilized due to its various assay panels targeted towards different diseases [18]. The continued advancement of proteomics technologies will expand our understanding of diseases, identify clinically valuable biomarkers, and ultimately contribute to human health. Notably, Olink technology has been successfully applied in studies involving CADASIL patients [19].

Our study addresses significant gaps in our understanding of CADASIL, particularly regarding the identification of plasma biomarkers associated with disease progression and underlying pathophysiology. While previous research has explored various aspects of CADASIL pathology, such as genetic mutations and vascular pathology, few studies have comprehensively investigated the proteomic landscape using advanced technologies like the Olink proteomics platform. Building upon this, our study aimed to characterize the proteomic profile of CADASIL patients and identify potential plasma biomarkers implicated in disease progression. Through this novel biomarker screening approach, we aimed to shed light on the molecular mechanisms underlying CADASIL pathology and offer insights into targeted interventions and personalized treatment strategies. By addressing these gaps in knowledge, our study contributes to advancing our understanding of CADASIL and holds promise for improving patient outcomes.

## 2. Materials and Methods

### 2.1. Ethics Statement

This study followed the ethical recommendations outlined in the Declaration of Helsinki on Humans and in the Standards of Good Clinical Practice ICH E6. Additionally, the Hospital Research Ethical Committee approved the study (study code: S1900024; approved date: 15 May 2019), and written informed consent was obtained from all participants prior to performing study-related procedures. This manuscript adheres to the applicable Consolidated Standards for Reporting Trials guidelines.

### 2.2. Study Design and Patients

An observational and prospective study was conducted at Virgen Macarena University Hospital in Seville, Spain. Patients were selected from the cohort at the Autonomic Reference Unit in Minority Neurovascular Diseases at the same hospital. In our center, a genetic panel protocol has been established for hereditary cerebral small vessel diseases [20], including the description of new pathogenic mutations [21]. Inclusion criteria consisted of patients with a pathogenic mutation in NOTCH3 identified through genetic testing and having a first-degree relative without the mutation. Individuals who did not provide written informed consent were excluded from the study. To assess differential expression between CADASIL patients and their healthy first-degree relatives, we conducted a proteomic comparison study using the Olink platform.

### 2.3. Sample Collection

All patients attended our hospital for the extraction of a total of 5 mL of fasting venous blood in a tube with ethylenediaminetetraacetic acid (EDTA) anticoagulant (BD Vacutainer K2EDTA spray-coated tubes). After extraction, the tubes were centrifuged at 3500× *g* for 15 min, and the plasma was stored at −80 °C in the Hospital Biobank until proteomic analysis. The blood samples that were used for the cardiovascular biomarker assessment were collected on the same day as the clinical characteristics. Samples were not collected during the occurrence of the stroke.

To ensure consistency and minimize bias, sample handling, and processing procedures were meticulously followed. Upon collection, samples were immediately labeled with unique identifiers to maintain anonymity and tracked throughout the study. Samples were randomized during processing to prevent any systematic errors or batch effects. Additionally, blind assessments were employed during the analysis phase to mitigate potential biases. Technicians responsible for sample analysis were blinded to the clinical status of the samples, ensuring impartiality in data interpretation. Calibration across different assay panels was rigorously maintained through regular quality control checks and calibration procedures following the manufacturer’s guidelines. This ensured the accuracy and reliability of protein measurements across all samples and assay panels used in the study. Overall, these measures were implemented to uphold the robustness and integrity of the study’s findings, ensuring that any observed differences in protein expression levels were reflective of true biological variations rather than procedural artifacts.

### 2.4. Proteomic Analysis

Olink proteomics conducted the proteomic analysis at Uppsala University, Sweden, utilizing the Proximity Extension Assay (PEA) technology [22]. This multiplex immunoassay involves a pair of “probes” (oligonucleotide-labeled antibodies) to bind to the target protein in the sample. The resulting sequence is then detected and quantified via real-time polymerase chain reaction using the Fluidigm^®^ BioMark™ HD System. This high-capacity platform allows for the simultaneous detection of 96 analytes across all samples analyzed, thereby reducing analysis time while requiring only a minimal sample volume (1 μL of plasma).

The results were presented in Normalized Protein eXpression (NPX) units, which are logarithmically related to protein concentration but cannot be directly converted to absolute concentration units. A higher NPX value signifies a higher protein expression level. It is crucial to note that NPX values can only be compared within the same protein and among samples analyzed in the same project run.

To ensure data integrity, each sample was run in singleton, adhering to the manufacturer’s recommendation. Blood samples were processed in the same run and on the same plate to minimize both intra- and inter-assay variability. Additionally, four internal controls were spiked into every sample, while each sample plate included six required and two recommended external controls, each placed in separate wells. These measures were implemented to maintain consistency and accuracy throughout the analysis process.

A total of 552 proteins were measured by six Olink panels. Each panel consists of 92 biomarkers, of which 534 proteins were unique due to protein duplication across six panels (https://olink.com/products-services/target/last, accessed on 7 September 2023):

- The neurology panel constitutes the focus of our investigation. The proteins included are associated with neuronal function, neurodegenerative processes, and mechanisms underlying brain damage repair. It may offer invaluable insights into early disease detection, disease monitoring, and potential therapeutic targets.
- The two cardiovascular panels (CVD II and III) provides a panoramic view of the vascular landscape in CADASIL, probing into proteins crucial for vascular homeostasis, myocardial contractility, and hemostatic equilibrium. CVD III panel uses a 1:100 dilution of samples.
- The inflammation panel dissects the dynamic interplay between systemic and localized inflammatory responses within the brain, unraveling key inflammatory proteins’ roles in disease pathogenesis and offering potential therapeutic avenues.
- The Cardiometabolic panel scrutinizes the intricate web of proteins implicated in metabolic dysregulation, including dyslipidemia, diabetes, and hypertension, unraveling the intricate interplay between metabolic factors and CADASIL progression. This panel uses a 1:2025 dilution of samples.
- The Oncology II panel sheds light on the intriguing intersection between vascular complications and tumorigenic implications in CADASIL, exploring proteins implicated in cell growth, apoptosis, and tumorigenesis.

### 2.5. Preliminary Data Analysis

As a preliminary step, we developed data sample pre-processing procedures. Samples below the limit of detection (LOD) were managed as follows:

- Proteins with less than 30% of samples below LOD or missing value per group (i.e., less than 3 individuals per group) were included in the differential expression analysis.
- Proteins with more than 30% of samples below LOD or missing per group and less than 70% of samples below LOD or missing value overall were considered possible on-off proteins. These proteins might show activation or detection based on patient characteristics within each group. Although excluded from the main analysis, an alternative contrast is performed to check whether there is evidence of missing or under LOD values association with one of the two groups with the Fisher Exact Test.
- Proteins with more than 70% of samples below LOD were excluded.

A quality control warning on the Neurology panel was produced on a control patient. The warning points to high variability among NPX replicas. Due to the reduced sample size, we kept the sample despite the issue. As a sensitivity analysis, we conducted an additional differential expression analysis without the flagged sample.

### 2.6. Statistics

To study the relationship of the proteomic profile between CADASIL patients and control cases, we performed three different statistical methods and approaches.

At first, we conducted an Exploratory Principal Components Analysis (PCA) to understand the main sources of variability in the expression data. Then, we used Partial Least Squares–Discriminant Analysis (PLS-DA) to identify distinctive patterns between CADASIL patients and healthy individuals.

Second, to contrast whether the CADASIL group has a different average expression than controls across all measured proteins, an Empirical Bayes moderated T-test (Implemented in the R limma package [23] version 4.3.1) was employed. Furthermore, we used the R programming language to create a volcano plot to visualize the expression of proteins in plasma, generate a heatmap for cluster analysis, and calculate the Variable Importance of Projection (VIP) scores from the PLS-DA.

The moderated *t*-test combines information from all assays to enhance variability estimation. To manage the family-wise error rate (FWER) resulting from multiple tests, we tried adjusting *p*-values using the Holm–Bonferroni method. Even so, given the exploratory nature and limitations of our study, we present results and discussion with unadjusted *p* values. Nonetheless, any interpretation of unadjusted results as positive should acknowledge that statistical evidence arises in a multiple comparisons context.

For each comparison, a log2 Fold Change is estimated; results from contrasts are ranked by evidence of differential expression among groups (absolute value of T statistic) and displayed in tables accordingly. 

Third, we conducted a gene set enrichment analysis (GSEA) following the method outlined by Tancin Lambert et al. [24]. GSEA assesses whether a group of genes or proteins shows differential expression compared to the rest in a case–control study. The analysis was conducted on the Olink dataset against the Hallmark v.2023 database (h.all.v2023.1.Hs.symbols.gmt) initially [50 gene sets]. Gene sets that met the criteria of a false discovery rate (FDR) q-value < 0.25 and a Normalized (NOM) *p*-value < 0.05 after 1000 permutations were considered statistically significant. Furthermore, we utilized an online tool on the MSigDB website to export gene sets using specific terms related to pathways implicated in CADASIL, the Hallmark database, and the term “*NOTCH3*” [1015 gene sets]. These terms included pathways such as cell adhesion, extracellular matrix, misfolding control, autophagy, angiogenesis, and the transforming growth factor β (TGFβ) signaling pathway [25].

## 3. Results

In this study, we analyzed an independent case–control cohort consisting of 15 patients, with eight diagnosed with CADASIL and seven serving as controls, who were first-degree relatives. These individuals were selected from seven families. The majority of CADASIL diagnoses (seven out of eight) were made through family screenings, while one patient was diagnosed after experiencing a stroke, confirmed by radiological findings. Among the CADASIL patients, six had mutations in exon 4 of the NOTCH3 gene, while the remaining two had mutations in exon 11. Migraine was a prevalent symptom among all CADASIL cases, with two experiencing auras. Additionally, two patients had lacunar ischemic strokes at ages 49 and 60 years, respectively. Brain MRI was conducted in six out of the seven CADASIL cases, with one patient having a normal MRI at the age of 28. Conversely, control patients were asymptomatic. Brain MRI was not performed on control patients due to ethical considerations. The clinical–pathological characteristics of the patients are detailed in Table 1.

A total of 517 proteins were analyzed across the 15 subjects using the six Olink panels previously described. Our analysis revealed six protein alterations with unadjusted *p*-values under 0.05: MMP-10, RSPO1, PPY, PSP-D, CCL28, and FGF-19. The complete list can be found in Table 2 (Complete table in Supplemental Material Table S1). Additionally, significant changes in fold change were observed, with RSPO1 and FGF-19 proteins showing increased levels, while PPY protein exhibited decreased levels.

**Figure 1:**
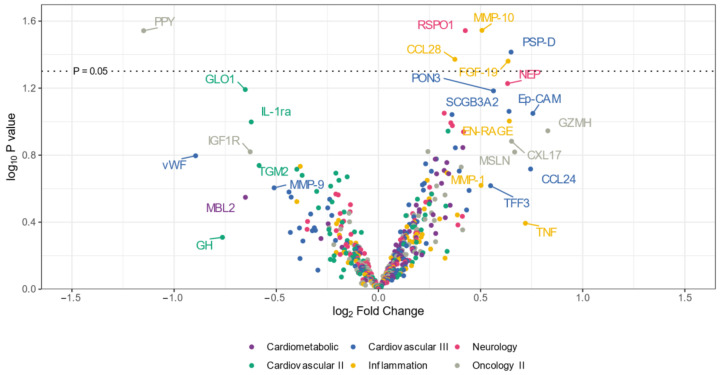
Volcano plot illustrating differentially expressed proteins in serum from CADASIL and healthy patients. Proteins on the positive (**right**) side indicate higher expression in CADASIL patients, whereas those on the negative (**left**) side suggest a higher presence in controls. The vertical position on the *y*-axis correlates with the statistical evidence of such relative abundance. Colors indicate the Olink panel.

**Figure 2:**
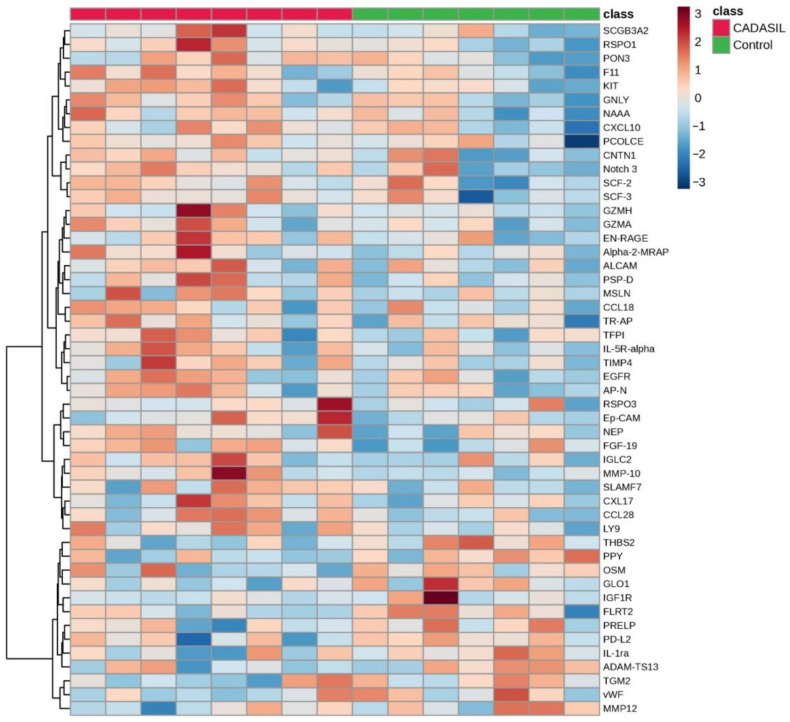
The heatmap illustrates the distribution of proteins identified as discriminative among CADASIL and control patients. Each row represents a protein, and each column represents a patient. Color intensity indicates the relative expression or abundance level of each protein across samples, with warmer colors indicating upregulated protein expression, while cooler colors indicate downregulated protein expression.

The volcano plot (Figure 1) illustrates that the expression levels of five proteins (CCL28, RSPO1, MMP-10, PSP-D, and FGF-19) were elevated in the CADSIL group compared to healthy individuals, while PPY protein showed decreased expression. Conversely, heatmap analysis (Figure 2) identified 50 differentially expressed proteins. Specifically, 37 proteins, including NOTCH3, PSP-D, GZMA, FGF-19, RSPO1, and CCL28, were found to be concentrated in CADASIL patients. On the other hand, 13 proteins such as PPY, IGF1R, THBS2, and IL-1ra were decreased compared to healthy patients.

### 3.1. PLS-DA Analysis

**Figure 3:**
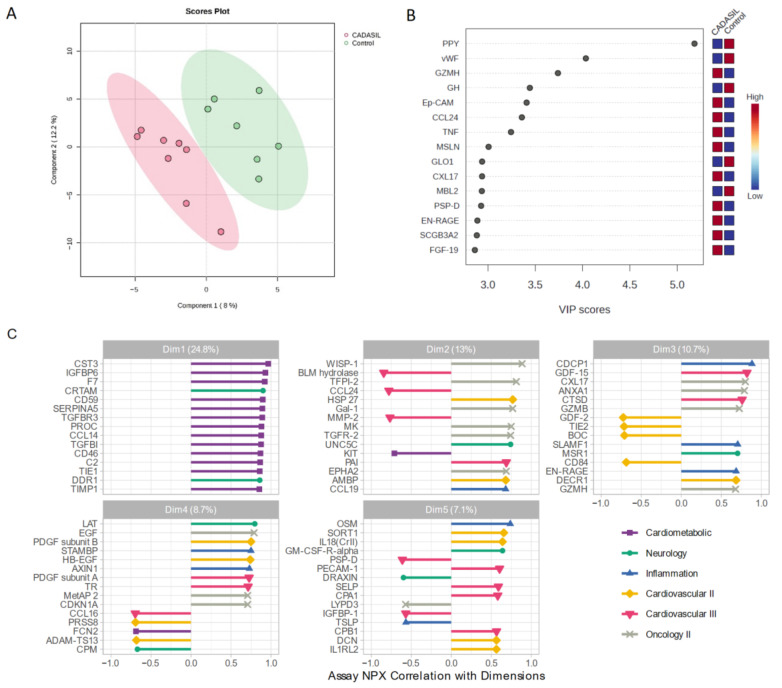
(**A**) Scores plot showcasing the distribution of samples in the space of the first two principal components (PC1 and PC2), with each point representing an observation from the study. Colors denote different groups, while ellipses illustrate the dispersion within each group. The variance explained by each component is indicated on the axes. (**B**) Variable Importance in Projection rank-score of the top 15 quantified proteins. Colored boxes on the right indicate the relative concentrations of the corresponding protein in CADASIL and control patients. (**C**) Depicts the correlation of Normalized Protein eXpression (NPX) with dimension and the top 15 correlated proteins, aiding in the interpretation of Principal Component dimension. Colors indicate the Olink panel.

We employed a PLS-DA approach to explore variability between CADASIL and control patients (Figure 3). This supervised analysis enabled the selection of the most discriminative proteins in the data to classify the samples. Figure 3A illustrates a clear separation of the control group from CADASIL patients, which suggests good discrimination based on the analyzed variables. The proteins that are significantly varying between the CADASIL and healthy patients were identified using the VIP scores of PLS-DA data. The top 15 proteins that distinguish the characteristic proteomic profiles of CADASIL and control were ranked as per the VIP score (Figure 3B). We conducted NPX analysis utilizing a Principal Component Analysis (PCA) method to compare serum protein profiles between CADASIL patients and healthy individuals (Figure 3C). The figure depicts NPX values for various proteins within the first five domains. It specifically focuses on the initial domain housing proteins associated with the cardiometabolic profile. Notably, CST3 is highlighted as the primary protein within this domain.

### 3.2. GSEA Analysis

In CADASIL patients, three gene sets were significantly upregulated according to the Hallmark v.2023 database after pre-ranked analysis. These sets were related to immune categories, specifically involving complement (ITGAN and Granzima A and B), allograft rejection (IL-13, TNF, and Granzima A), and inflammatory response (CCL24, CD70, and SLAMF1). Conversely, one gene set was significantly downregulated in CADASIL patients, related to the development category, specifically epithelial–mesenchymal transition (TGM2, TNFRSF12A, and PTX3).

**Figure 4:**
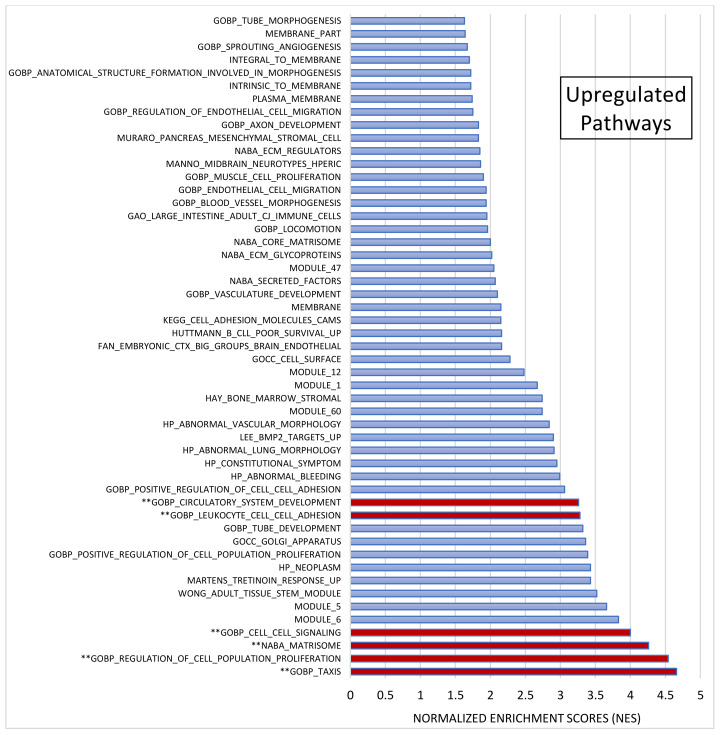
Gene set enrichment analysis (GSEA). Out of 1015 selected gene sets, 155 remained significant following pre-ranked analysis. The bar chart displays absolute Normalized Enrichment Scores (NES) significantly differing between CADASIL and control patients. False Discovery Rate (FDR) q-value < 0.25 and Nominal (NOM) *p* value < 0.05): 51 gene sets were identified as upregulated. Pathways double marked (**) were further analyzed.

**Figure 5:**
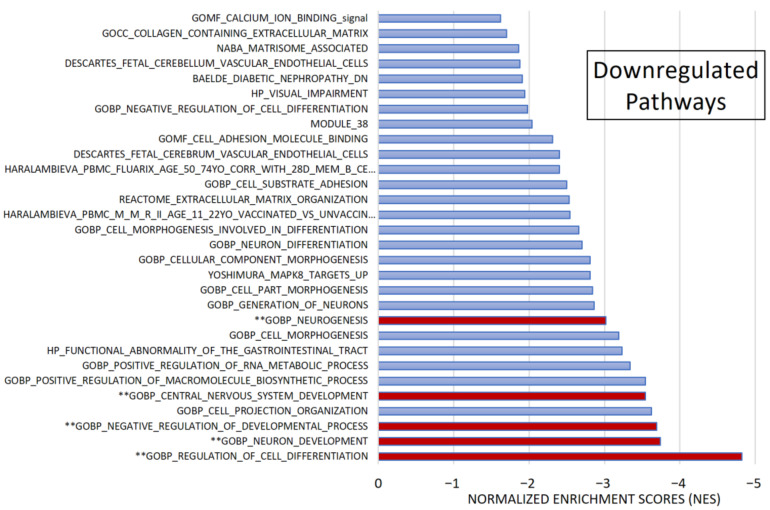
Gene set enrichment analysis (GSEA). Among 1015 selected gene sets, 155 remained significant following pre-ranked analysis. The bar chart displays absolute Normalized Enrichment Scores (NES) significantly differing between CADASIL and control patients. False Discovery Rate (FDR) q-val < 0.25 and Nominal (NOM) *p* value < 0.05): 30 gene sets were identified as downregulated. Pathways marked (**) were further analyzed.

After pre-ranked analysis, 155 gene sets remained: 51 gene sets showed significant upregulation in CADASIL patients (Figure 4), and 30 gene sets showed significant downregulation (Figure 5).

Among the most significant pathways upregulated in CADASIL, six pathways stand out for their high NES (Figure 4): Taxis, Regulation of cell population proliferation, Matrisome, Cell–cell Signaling, Leukocyte cell–cell adhesion, and Circulatory system development. Conversely, the five most significant pathways for downregulated genes were selected (Figure 5): Regulation of cell differentiation, Neuron development, Negative regulation of developmental processes, Central nervous system development, and Neurogenesis.

**Figure 6:**
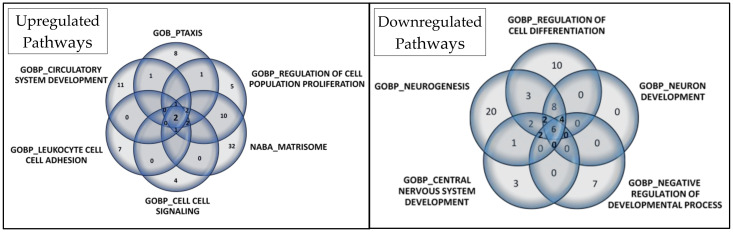
Venn diagrams depict the core genes selected in gene set enrichment analysis for upregulated and downregulated pathways. In the Venn diagram for the 6 selected upregulated gene sets, 2 proteins overlapped, while in the Venn diagram for the 5 selected downregulated gene sets, 6 proteins overlapped.

A Venn diagram visually represents overlapped proteins in each set (Figure 6). In upregulated pathways, Annexin A1 (ANXA1) and IL-6 were consistently found. Additionally, six proteins were present in five of the six selected gene sets: C-C motif chemokine 24 (CCL24), C-C motif chemokine 5 (CCL5), C-X-C motif chemokine 10 (CXCL10), Interleukin-10 (IL10), Tumor necrosis factor (TNF), and Lymphotactin (XCL1). These findings are detailed in Table S2.

Regarding downregulated pathways, six proteins overlapped: Cadherin-1 (CDH1), Receptor tyrosine-protein kinase erbB-2 (ERBB2), Leptin (LEP), Tyrosine-protein kinase Lyn (LYN), Microtubule-associated protein tau (MAPT), and Receptor-type tyrosine-protein phosphatase S (PTPRS). Additionally, eight proteins were present in four of the five selected gene sets: Amphiregulin (AREG), Delta-like protein 1 (DLL1), Kallikrein-6 (KLK6), NAD-dependent protein deacetylase sirtuin-2 (SIRT2), Superoxide dismutase [Cu-Zn] (SOD1), Superoxide dismutase [Mn], mitochondrial (SOD2), Tumor necrosis factor receptor superfamily member 21 (TNFRSF21), and Vascular endothelial growth factor A (VEGFA). These details are provided in Table S3.

### 3.3. CADASIL Patients

**Figure 7:**
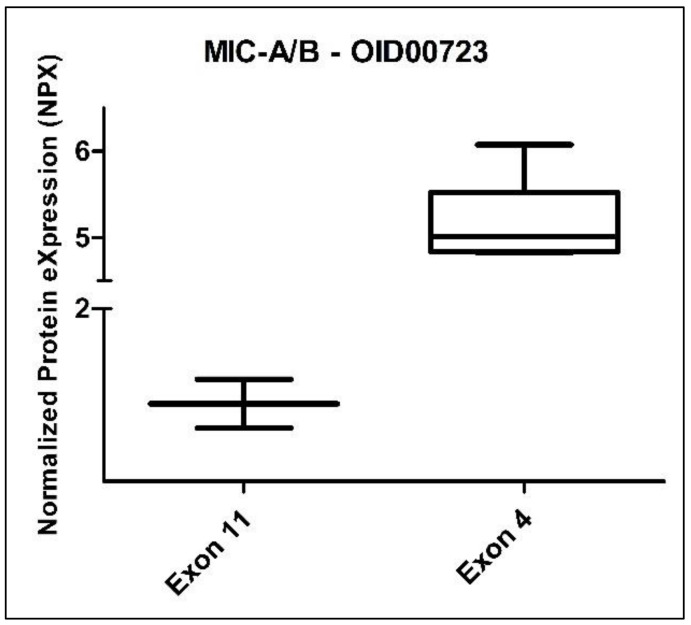
Box plot of the MIC-A/B protein (Oncology II Olink panel) in CADASIL patients, divided according to exon: exon 11 (2 subjects) and exon 4 (6 subjects).

When we analyzed the group of eight CADASIL patients stratified by gender (three female vs. five male), we did not observe any statistically significant differences among the 517 evaluated proteins. However, when we divided these patients by exon (six from exon 4 vs. two from exon 11), we identified a statistically significant difference in the protein MIC-A/B (MHC class I polypeptide-related sequence A/B) from the Oncology panel. The Benjamini–Hochberg adjusted *p*-value for MIC-A/B was 0.001972 (Figure 7). It is worth noting that the comparison between exons corresponded to mutations classified as high-risk and medium-risk, according to Hack et al. [26].

## 4. Discussion

This comprehensive analysis of an independent case–control cohort has unveiled significant statistical differences, particularly in fold-change measurements. Notably, RSPO1 and FGF-19 proteins exhibited increased levels, while PPY protein displayed decreased levels. Furthermore, our findings suggest the importance of specific proteins and pathways in CADASIL pathophysiology. The identification of MIC-A/B protein as a distinguishing factor between high- and medium-risk NOTCH3 gene mutations highlights its potential in stratifying patients by mutation severity. To our knowledge, this study is the first to investigate differences in proteomic profiles between CADASIL patients and control cases. These findings underscore the intricacy of CADASIL and emphasize the need for further research to fully comprehend its underlying mechanisms.

This work included eight patients diagnosed with CADASIL, six of whom exhibited pathogenic mutations primarily concentrated in exon 4 of the NOTCH3 gene, while the remaining two patients had mutations within the more common range, specifically within exon 11. All these missense mutations are classified as pathogenic in ClinVar, a database of human genetic variations and interpretations. Moreover, according to Hack et al., patients with mutations in exon 11 should be classified as medium-risk and exhibit milder and less frequent symptoms compared to those with mutations in exon 4, who are categorized as high-risk [26]. Recent research has emphasized a correlation between mutations in the epidermal growth factor-like (EGFr) repeat domains and disease severity. Specifically, NOTCH3cys variants located in EGFr domains 1 to 6 have been associated with a higher risk of stroke and increased white matter hyperintensity volume compared to variants in EGFr domains 7 to 34 [26]. These findings align closely with our clinical observations. In fact, among the eight CADASIL patients, those who experienced strokes had mutations in exon 4, particularly within EGFr 3 and 5. However, patients with mutations in exon 11, specifically EGFr 14, showed a milder clinical phenotype. This further underscores the importance of genetic stratification in CADASIL prognosis. Beyond the NOTCH3cys EGFr group, cardiovascular risk factors and sex have been shown to influence CADASIL disease severity. Notably, male sex, hypertension, diabetes, and smoking have been associated with various neuroimaging markers and stroke occurrence in CADASIL patients [27]. In our cohort, cardiovascular risk factors distribution was largely similar, except for smoking, which was more prevalent among CADASIL patients. Moreover, both stroke patients were males, smokers, and had dyslipidemia, with one also exhibiting hypertension and diabetes, consistent with previous literature. However, the limited sample size precludes definitive conclusions regarding these correlations. In our proteomic study, which compared genetically diagnosed CADASIL patients to healthy, genetically related controls, we explored numerous protein markers potentially associated with the disease. Among these markers, significant fold changes were observed. RSPO1, known for its role in vascular homeostasis and angiogenesis [28], exhibited increased levels, suggesting its potential involvement in CADASIL-related vascular pathology. Additionally, recent research has relatively strongly associated RSPO1 with general fluid cognitive ability [29], indicating its broader implications beyond vascular function. FGF-19, implicated in metabolic regulation and potential implications in oxidative stress management [30], also showed elevated levels, hinting at its role in metabolic dysregulation often observed in CADASIL, a disease with known vascular and metabolic disturbances. Moreover, FGF-19 appeared to be positively associated with ischemic stroke [31], further emphasizing its relevance in cerebrovascular diseases. Conversely, PPY, also known as pancreatic polypeptide Y, serves as a hormone secreted by the pancreas, playing vital roles in appetite regulation and energy metabolism. Interestingly, studies have suggested that various plasma proteins, including PPY, are associated with amyloid burden in the brain [32], indicating a potential link between PPY dysregulation and neurodegenerative processes. The observed downregulation of PPY in CADASIL patients suggests a potential dysfunction in metabolic regulatory mechanisms associated with this condition. These findings underscore the intricate interplay between metabolic dysregulation and neurological pathology in CADASIL, warranting further investigation into the mechanistic underpinnings of these protein alterations.

Similar results were obtained through a volcano plot analysis, revealing significant differences in the expression of CCL28, RSPO1, MMP-10, PSP-D, FGF-19, and PPY proteins in CADASIL patients. These proteins, encompassing diverse roles in biological processes such as immune regulation, cell proliferation, tissue remodeling, and metabolism, were observed to be significantly upregulated in CADASIL patients. This upregulation may suggest their involvement in the pathophysiology of the disease. Consistent with previous findings, PPY was downregulated. Interestingly, previous studies have indicated that proteins related to the coagulation process, particularly the von Willebrand factor, have been associated with CSVD and white matter hyperintensity burden [19]. However, in our volcano plot analysis and heatmap, proteins such as the von Willebrand factor were decreased in CADASIL patients compared to healthy patients. This discrepancy underscores the complexity of CADASIL pathology and the need for further investigation into the underlying mechanisms driving these differential protein expressions.

Curiously, NPX analysis revealed a significant difference in protein expression between CADASIL patient groups and healthy patients, particularly in the first domain, which is intriguing. Notably, the elevated expression of CST3 in patients with CADASIL suggests a potential association with this disease. CST3, also known as cystatin C, is a protein primarily produced by nucleated cells and is involved in the regulation of protease activity. Recent studies have implicated CST3 dysregulation in various neurological disorders, including Alzheimer’s disease and cerebral small vessel disease, which shares pathological similarities with CADASIL [33]. CST3 is also known to be expressed in cerebrospinal fluid and has been proposed as a biomarker for neurodegenerative diseases due to its association with neuronal injury and neuroinflammation [34].

The implementation of GSEA yielded invaluable insights into pathways pertinent to CADASIL pathology. In our primary GSEA, while no significant pathways emerged, a secondary pre-ranked analysis highlighted several intriguing pathways. Notably, the complement pathway emerged prominently, consistent with previous research linking it to CADASIL [13,35]. Activation of this pathway has been observed in VSMCs harboring the Arg133Cys mutation, concurrent with elevated levels of pro-inflammatory markers such as IL-6 and ICAM-1 [13]. Moreover, these proteins have been linked to the burden of radiological markers of CSVD [36]. Our proteomic analysis corroborated these findings, demonstrating raised IL-6 and ICAM-1 levels in the complement pathway and the inflammatory response pathway, suggesting their potential as biomarkers for further investigation.

Further exploration via GSEA revealed the upregulation of cytokines and growth factors, underscoring their relevance in CADASIL. ANXA1, identified as significant across various pathways, holds particular promise due to its pivotal role in inflammation [37]. Similarly, CXCL10, elevated in our study, has been implicated in neuroinflammation and neuronal injury in cerebral arteriopathy [38]. Conversely, downregulated pathways unveiled insights into CADASIL pathogenesis. VEGFA, a potent proangiogenic factor, was diminished, consistent with reduced cerebral blood vessel density observed in CADASIL [39]. Notably, PTPRS downregulation aligns with recent findings implicating the PTPRS gene in CADASIL-related vascular damage [40].

In addition to these pathways, other proteins emerged as potential markers in our study. COL18A1, identified in upregulated pathways, aligns with previous research demonstrating its increased levels in CADASIL mouse models and postmortem vessels [41]. Similarly, AREG downregulation suggests a potential mechanism contributing to inflammation in CADASIL, given its role in cytokine suppression [42,43]. Reduced SOD1 expression, observed in our study, concurs with previous reports of decreased SOD1 levels in CADASIL VSMCs, implicating oxidative stress in disease pathology [44]. Contrary to expectations, TIMP3 and VTN did not emerge as significant in our analysis despite previous associations with CADASIL pathology [45].

The discovery of heightened MIC-A/B expression in CADASIL patients with mutations in exon 4 offers intriguing insights into potential immunological mechanisms driving the disease. MIC-A/B molecules play a pivotal role in immune surveillance by interacting with natural killer cells and cytotoxic T cells, thereby regulating immune responses against infected or stressed cells [46]. The observed variance in MIC-A/B expression between CADASIL patients with mutations in exon 4 compared to those in exon 11 suggests a nuanced relationship between specific genetic variants and immune dysregulation in CADASIL pathogenesis. This finding prompts further investigation into the interplay between NOTCH3 mutations and immune responses, illuminating the intricate relationship between vascular pathology and immune activation in CADASIL. Understanding the impact of exon-specific mutations on immune modulation may provide valuable insights into disease progression. This can inform the development of targeted therapeutic strategies aimed at alleviating immune-mediated damage in CADASIL patients. Notably, MIC-A/B, a protein previously unexplored in CADASIL literature, has been associated with an inflammatory state in diabetic patients and linked to atherosclerotic lesions [45,47]. Recent studies have identified elevated MIC-A/B levels in stroke patients with carotid atherosclerotic plaques, suggesting a potential contribution of NK cells to plaque instability [46,48]. Given that mutations in exon 4 confer a higher risk of earlier stroke onset, the increased expression of MIC-A/B in these patients may be indicative of greater plaque instability or lesion formation preceding stroke in CADASIL. Consequently, further analysis of this protein could yield valuable insights into CADASIL pathophysiology.

The primary strength of this study lies in its comprehensive approach to characterizing the proteomics of patients with CADASIL. It employs a carefully selected case–control cohort with familial controls to minimize the influence of confounding genetic factors not related to CADASIL. Furthermore, detailed genetic analysis reveals specific mutations in the NOTCH3 gene, further supporting the validity of the studied sample. Secondly, the study employs six Olink panels, encompassing a broad range of proteins, allowing for a comprehensive analysis of the proteome. Additionally, the application of multiple analytical techniques, such as protein analysis and GSEA, provides a comprehensive view of the biological alterations associated with CADASIL, enriching our understanding of the underlying mechanisms of the disease. However, several limitations should be acknowledged, including the relatively small sample size and lack of replication in an independent cohort, which limits the generalizability of the findings and the power to detect subtle changes. The cross-sectional design precludes establishing cause-and-effect relationships between protein alterations and CADASIL development. Moreover, the study does not explore protein–protein interactions, which is potentially crucial for understanding disease mechanisms. Furthermore, the lack of statistical significance in certain analyses suggests the need for future studies with larger samples to validate and generalize the findings.

While we have acknowledged the limitations associated with sample size in our study, it is imperative to delve deeper into potential biases that may affect the generalizability of our results. One such concern is selection bias, which could arise due to the recruitment of patients from a specific demographic or clinical setting. In our case, patients were recruited from a single medical center, potentially limiting the representativeness of our findings to the broader CADASIL population. Future studies could address this limitation by employing multicenter collaborations or population-based cohorts to ensure greater diversity and generalizability of results. Additionally, efforts to minimize selection bias through rigorous inclusion and exclusion criteria should be emphasized in future research endeavors. Moreover, the lack of replication in an independent cohort underscores the need for collaborative efforts across research institutions to validate and corroborate our findings. By pooling data from multiple cohorts, future studies can enhance the robustness and reliability of proteomic analyses in CADASIL. These initiatives will not only strengthen the evidence base but also facilitate the translation of research findings into clinical practice, ultimately benefiting CADASIL patients worldwide.

In future studies, it will be crucial to validate the biomarkers identified in this study through independent replication in larger cohorts of CADASIL patients. Additionally, exploring the mechanistic roles of these biomarkers in disease progression could provide insights into novel therapeutic targets. For example, further investigation into the involvement of RSPO1 and FGF-19 in CADASIL-related vascular pathology may uncover potential therapeutic interventions targeting angiogenesis and oxidative stress management. Moreover, understanding the impact of PPY downregulation on metabolic dysregulation in CADASIL could inform strategies aimed at restoring metabolic balance. Finally, exploring the immunological mechanisms underlying the observed alterations in MIC-A/B expression may offer opportunities for immune-modulating therapies to mitigate disease progression. By addressing these research avenues, future studies can contribute to a deeper understanding of CADASIL pathophysiology and facilitate the development of effective therapeutic interventions.

We presented unadjusted *p*-values to provide transparency regarding statistical significance testing and to facilitate comparisons with future studies. However, it is important to acknowledge the potential inflation of Type I error rates due to multiple testing. Therefore, the interpretation of statistically significant results should be tempered by an awareness of this limitation. Future studies with larger sample sizes and stringent statistical corrections will be necessary to validate our findings and elucidate the true significance of the observed protein alterations in CADASIL pathophysiology. Nonetheless, our exploratory analysis serves as an important starting point for generating hypotheses and guiding future research efforts aimed at understanding the molecular mechanisms underlying CADASIL.

## 5. Conclusions

In our research, significant differences and fold changes were identified in the proteins we investigated, as supported by volcano plot analysis. Through this analysis, we observed noteworthy alterations in RSPO1, FGF-19, and PPY proteins. While we could not definitively establish the discriminatory potential of any measured proteins between CADASIL patients and controls, the use of GSEA provided vital insights into correlated biological pathways. Specifically, pathways associated with complement activation and inflammatory responses emerged as significant in CADASIL patients. Proteins like ANXA1, IL-6, and ICAM-1 were implicated, emphasizing the need to consider cytokines, growth factors, and other notable proteins such as CXCL10 and COL18A1 in future investigations. Conversely, our study revealed decreased expression of proteins like VEGFA, PTPRS, and AREG in CADASIL patients, aligning with prior research. However, the challenge of recruiting eligible CADASIL subjects has severely limited sample sizes, highlighting the necessity for larger multicenter studies with well-defined inclusion criteria. Additionally, it is crucial that future research prioritizes identifying and validating specific proteins with robust diagnostic potential in CADASIL, potentially through exploring combinations of multiple markers to enhance diagnostic accuracy. This approach could facilitate the development of targeted therapeutic strategies aimed at mitigating immune-mediated damage, ultimately leading to improved clinical outcomes for CADASIL patients.

## Figures and Tables

**Table 1:** Characteristics of patients included in the analysis are divided into two groups: CADASIL (N = 8) and control by first-degree relatives (N = 7).

	CADASIL	Control
n	8	7
Female (%)	3 (37.5)	3 (42.9)
Median age (range)	46.5 (28–64)	45 (21–58)
NOTCH3 mutation (n)		
Exon 4 (*n*)	c.619C>T (p.Arg207Cys) (3) c.397C>T (p.Arg133Cys) (2) c.544C>T (p.Arg182Cys) (1)	-
Exon 11 (*n*)	c.1672C>T (p.Arg558Cys) (2)	-
Comorbidities		
Smoking (%)	4 (50)	0 (0)
Hypertension (%)	3 (37.5)	3 (42.9)
Diabetes Mellitus (%)	1 (12.5)	2 (28.6)
Dyslipidemia (%)	3 (37.5)	3 (42.9)
BMI > 25 (%)	3 (37.5)	2 (28.6)
Neurological manifestations		
Migraine (%)	8 (100)	0 (0)
Cognitive impairment (%)	2 (25)	0 (0)
Ischemic stroke *(%)*	2 (25)	0 (0)
Median Fazekas score on brain MRI	2	-
White matter hyperintensities on brain MRI (%)	5 (85.7)	-

CADASIL: Cerebral Autosomal Dominant Arteriopathy with Subcortical Infarcts and Leukoencephalopathy; N: number of subjects; MRI: magnetic resonance imaging; BMI: body mass index; Arg: Arginine; Cys: Cysteine.

**Table 2:** Top 6 protein assay differences between CADASIL patients and controls, ranked according to T-statistic absolute value. Positive log2 Fold-Change (FC) estimates indicate how many times the expression is doubled in CADASIL patients relative to controls, while negative values indicate the times doubling in control patients. CI.L and CI.R represent the 95% Confidence Interval bounds, showing a range of compatible log2 FC estimates. Adjusted *p*-values were calculated using the Holm–Bonferroni method.

Assay	Panel	UniProt	log_2_FC	CI.L	CI.R	t	*p* Value	adj.P.Val
MMP-10	Inflammation	P09238	0.506696	0.060209	0.953183	2.400093	0.028521	1
RSPO1	Neurology	Q2MKA7	0.425335	0.050259	0.80041	2.398288	0.028624	1
PPY	Oncology II	P01298	−1.14913	−2.16279	−0.13547	−2.39754	0.028667	1
PSP-D	Cardiovascular III	P35247	0.649328	0.038917	1.259739	2.249735	0.038452	1
CCL28	Inflammation	Q9NRJ3	0.374214	0.014277	0.734151	2.198787	0.04249	1
FGF-19	Inflammation	O95750	0.634688	0.020695	1.248682	2.186181	0.043548	1

## Data Availability

The original contributions presented in the study are included in the article/Supplementary Materials, further inquiries can be directed to the corresponding author.

## Supplementary Materials

The following supporting information can be downloaded at https://www.mdpi.com/article/10.3390/jcm13113138/s1. Table S1. Table with top 15 protein assay differences among groups, results are ranked according to T-statistic absolute value. Table S2. Proteins listed in the 6 most significant upregulated pathways related to CADASIL from GSEA analysis (1015 gene sets). Table S3. Proteins listed in the 5 most significant downregulated pathways related to CADASIL from GSEA analysis (1015 gene sets).
