# Supplementary material for: A Search for New Biological Pathways in Cerebral Autosomal Dominant Arteriopathy with Subcortical Infarcts and Leukoencephalopathy by Proteomic Research
Source: J Clin Med. 2024 May 27;13(11):3138. doi: 10.3390/jcm13113138 (PMC11172732; doi:10.3390/jcm13113138)
Supplement: Supplementary file 1 [file jcm-13-03138-s001.zip › jcm-2976172-supplementary.pdf]

Supplement

Table S1. Top 15 protein assay differences among groups, ranked according to T-statistic absolute value. Positive log_2 Fold-Change (FC) estimates indicate the degree of expression doubling in CADASIL patients relative to controls, while negative values represent the degree of doubling in control patients. CI.L and CI.R represent the 95% Confidence Interval bounds for compatible log_2 FC estimates. Adjusted p-values were calculated using the Holm-Bonferroni method.

Assay	Panel	UniProt	log_2FC	CI.L	CI.R	t	P.Value	adj.P.Val
MMP-10	Inflammation	P09238	0,506696	0,060209	0,953183	2,400093	0,028521	1
**RSPO1**	Neurology	Q2MKA7	0,425335	0,050259	0,80041	2,398288	0,028624	1
**PPY**	Oncology II	P01298	-1,14913	-2,16279	-0,13547	-2,39754	0,028667	1
PSP-D	Cardiovascular III	P35247	0,649328	0,038917	1,259739	2,249735	0,038452	1
CCL28	Inflammation	Q9NRJ3	0,374214	0,014277	0,734151	2,198787	0,04249	1
**FGF-19**	Inflammation	O95750	0,634688	0,020695	1,248682	2,186181	0,043548	1
NEP	Neurology	P08473	0,632256	-0,02763	1,292144	2,026342	0,05923	1
GLO1	Cardiovascular II	Q04760	-0,65167	-1,34684	0,043508	-1,98254	0,064345	1
PON3	Cardiovascular III	Q15166	0,563175	-0,04054	1,166895	1,972865	0,065527	1
SCGB3A2	Cardiovascular III	Q96PL1	0,639255	-0,10328	1,381794	1,820724	0,086856	1
NAAA	Neurology	Q02083	0,32208	-0,05487	0,699029	1,807053	0,089045	1
Ep-CAM	Cardiovascular III	P16422	0,755904	-0,12965	1,641457	1,805269	0,089334	1
Notch 3	Cardiovascular III	Q9UM47	0,360143	-0,06382	0,784105	1,796541	0,090761	1
EN-RAGE	Inflammation	P80511	0,640613	-0,13484	1,416069	1,747139	0,09922	1
IL-1ra	Cardiovascular II	P18510	-0,62251	-1,37899	0,133984	-1,74032	0,100439	1

Table S2. Proteins listed in the **6 most significant upregulated pathways** related to CADASIL from Gene set Enrichment Analysis (1015 gene sets). The following are ranked alphabetically in decreasing order according to their presence in more or fewer gene sets. The total number of proteins in each gene set is shown at the bottom of the table. **Gene set 1**: GOBP TAXIS, **Gene set 2**: GOBP REGULATION OF CELL POPULATION PROLIFERATION, **Gene set 3**: NABA MATRISOME, **Gene set 4**: GOBP CELL CELL SIGNALING, **Gene set 5**: GOBP LEUKOCYTE CELL CELL ADHESION, and **Gene set 6**: GOBP CIRCULATORY SYSTEM DEVELOPMENT.

		GENE SET					
	**PROTEIN**	**1**	**2**	**3**	**4**	**5**	**6**
1	**ANXA1**	x	x	x	x	x	x
2	**IL6**	x	x	x	x	x	x
3	CCL24	x	x	x	x		x
4	CCL5	x	x	x	x	x	
5	**CXCL10**	x	x	x	x		x
6	IL10	x	x	x		x	x
7	TNF		x	x	x	x	x
8	XCL1	x	x	x	x	x	
9	ABL1		x		x	x	x
10	CCL11	x	x	x			x
11	CXCL11	x	x	x	x		
12	CXCL13	x		x	x		x
13	CXCL5	x	x	x	x		
14	CXCL9	x	x	x	x		
15	GDNF	x	x	x	x		
16	IL2		x	x	x	x	
17	IL4	x	x	x		x	
18	KIT	x	x		x		x
19	LIF		x	x	x		x
20	NOTCH1	x	x		x		x
21	NRP1	x	x		x		x
22	PGF		x	x	x		x
23	VCAM1	x	x			x	x
24	VEGFD	x	x	x			x
25	ANGPT1		x	x			x
26	CCL14	x	x	x			
27	CCL16	x		x	x		
28	CCL18	x		x	x		
29	CCL20	x		x	x		
30	CCL28	x		x		x	
31	CD70		x		x	x	
32	COL1A1			x	x		x
33	COMP		x	x			x
34	CXCL1	x	x	x			
35	DPP4	x	x			x	
36	EGFR		x		x		x
37	HGF			x	x		x
38	IGFBP6		x	x	x		
39	LILRB1		x		x	x	

		GENE SET					
	**PROTEIN**	**1**	**2**	**3**	**4**	**5**	**6**
41	NTRK2		x		x		x
42	NTRK3	x	x				x
43	SLAMF1	x	x			x	
44	SMOC2	x		x			x
45	SPARC		x	x			x
46	TNC		x	x	x		
47	VEGFA		x	x			x
48	ADA		x			x	
49	ANG		x				x
50	ANGPTL3			x			x
51	AREG		x	x			
52	CD38		x		x		
53	CD40		x				x
54	CD46		x			x	
55	CD6		x			x	
56	CDH3		x		x		
57	CDH5		x				x
58	**COL18A1**		x	x			
59	CPE				x		x
60	CRTAM		x			x	
61	DRAXIN	x			x		
62	EFNA4	x			x		
63	ENG		x				x
64	ERBB3		x				x
65	ERBB4		x				x
66	FAP		x				x
67	GAS6		x	x			
68	GRN		x				x
69	IGFBP3		x	x			
70	IL13		x	x			
71	IL16	x		x			
72	ITGAV		x				x
73	PLXNB2	x		x			
74	PLXNB3		x	x			
75	REG1A		x	x			
76	ROBO2	x					x
77	RSPO3				x		x
78	SOD2		x				x
79	ST6GAL1	x	x				

40	LILRB2		x		x	x	

80	TGFBI			x			x

		GENE SET					
	**PROTEIN**	**1**	**2**	**3**	**4**	**5**	**6**
81	TGFBR3		x				x
82	TIE1		x				x
83	TIMP1		x	x			
84	TNFRSF13B		x			x	
85	TNFSF13		x	x			
86	TNFSF13B		x	x			
87	UMOD		x			x	
88	ACE2		x				
89	ALCAM	x					
90	ARTN			x			
91	CCL15			x			
92	CCL19			x			
93	CD27					x	
94	CD4					x	
95	CD5					x	
96	CEACAM1						x
97	CHI3L1						x
98	CHL1	x					
99	CNTN1	x					
100	CST3			x			
101	CST5			x			
102	CSTB			x			
103	CTSC			x			
104	CTSS			x			
105	CTSV			x			
106	CTSZ			x			
107	CXCL16	x					
108	DDR1		x				
109	DEFA1	x					
110	DLL1						x
111	EFEMP1			x			
112	EPHA2						x
113	EPHB4						x
114	FCN2			x			
115	**ICAM1**					x	
116	IL1RL2					x	
117	ITGAM				x		
118	MATN3			x			
119	MB						x
120	MEGF9			x			

		GENE SET					
	**PROTEIN**	**1**	**2**	**3**	**4**	**5**	**6**
121	MEPE			x			
122	MERTK				x		
123	MET	x					
124	MFAP5			x			
125	MMP7			x			
126	NCAM1	x					
127	NCAN			x			
128	NID1			x			
129	NRP2						x
130	NRTN			x			
131	OSMR		x				
132	PAM						x
133	PAPPA			x			
134	PCOLCE			x			
135	PI3			x			
136	PLA2G7	x					
137	PRCP						x
138	PRSS2			x			
139	REN				x		
140	RSPO1				x		
141	SELE					x	
142	SELL					x	
143	SERPINA12			x			
144	SERPINA5			x			
145	SERPINA7			x			
146	SPARCL1			x			
147	SPON1			x			
148	SPON2			x			
149	TCL1A		x				
150	THBS4						x
151	TIMP4			x			
152	TLR3						x
153	TSLP		x				
154	WFIKKN1			x			
	Total number of proteins in the gene set	43	75	80	41	29	54

Table S3. Proteins listed in the **5 most significant downregulated pathways** related to CADASIL from Gene set Enrichment Analysis (1015 gene sets). The following are ranked alphabetically in decreasing order according to their presence in more or fewer gene sets. The total number of proteins in each gene set is shown at the bottom of the table. **Gene set 1**: GOBP REGULATION OF CELL DIFFERENTIATION, **Gene set 2**: GOBP NEURON DEVELOPMENT, **Gene set 3**: GOBP NEGATIVE REGULATION OF DEVELOPMENTAL PROCESS, **Gene set 4**: GOBP CENTRAL NERVOUS SYSTEM DEVELOPMENT, and **Gene set 5**: GOBP NEUROGENESIS.

		GENE SET				
	**PROTEIN**	**1**	**2**	**3**	**4**	**5**
1	**CDH1**	x	x	x	x	x
2	**ERBB2**	x	x	x	x	x
3	**LEP**	x	x	x	x	x
4	**LYN**	x	x	x	x	x
5	**MAPT**	x	x	x	x	x
6	**PTPRS**	x	x	x	x	x
7	**AREG**	x	x	x		x
8	DLL1	x		x	x	x
9	KLK6	x	x		x	x
10	SIRT2	x		x	x	x
11	**SOD1**	x	x	x		x
12	SOD2	x	x	x		x
13	TNFRSF21	x	x		x	x
14	**VEGFA**	x	x	x		x
15	ADM	x	x			x
16	AXL	x			x	x
17	BOC	x	x			x
18	CCL3	x		x		x
19	CX3CL1	x	x			x
20	EPHB6		x		x	x
21	GAS6	x		x		x
22	IGF1R		x		x	x
23	IL33	x			x	x
24	NBL1	x	x			x
25	NRP2		x		x	x
26	PLXNB1	x	x			x
27	PLXNB3	x	x			x
28	PRTG	x		x		x
29	RET	x	x			x
30	SPOCK1		x		x	x
31	STK4	x		x	x	
32	TNXB	x	x			x
33	UNC5C		x		x	x
34	VIM		x		x	x
35	ARTN		x			x
36	AXIN1	x		x		
37	CCL17	x		x		
38	CEACAM1	x		x		
39	DDR1		x			x

		GENE SET				
	**PROTEIN**	**1**	**2**	**3**	**4**	**5**
48	SCARB2		x			x
49	SORT1	x		x		
50	TGM2	x				x
51	VWC2	x				x
52	ANXA1					x
53	AZU1					x
54	BCAN				x	
55	CCL11					x
56	CCL19	x				
57	CD160			x		
58	CD38					x
59	CDKN1A			x		
60	CNTN5					x
61	DCN			x		
62	EGF				x	
63	ERBB3					x
64	ERBB4					x
65	FADD	x				
66	FASLG			x		
67	GRN					x
68	HGF					x
69	IL2					x
70	IL5	x				
71	IL6					x
72	IL7	x				
73	IL7R	x				
74	MDGA1					x
75	MET					x
76	MSR1	x				
77	NID1	x				
78	NOTCH1					x
79	NRP1					x
80	NTRK2					x
81	NTRK3					x
82	OSM	x				
83	ROBO2					x
84	SPARC			x		
85	SRC				x	
86	THBS2			x		

40	DNER				x	x
41	FLRT2		x			x
42	GPC1	x				x
43	IL18	x		x		
44	MANF		x			x
45	PGLYRP1	x		x		
46	REG3A	x		x		
47	RGMA		x			x

87	THBS4			x		
88	THPO	x				
89	TNFSF14	x				
90	VCAM1					x
91	VEGFD					x
	Total number of proteins in the gene set	48	32	30	23	63
